# Prevalence of vertebral fractures in a disease activity steered cohort of patients with early active rheumatoid arthritis

**DOI:** 10.1186/1471-2474-13-125

**Published:** 2012-07-23

**Authors:** Linda Dirven, M van den Broek, J H L M van Groenendael, W M de Beus, P J S M Kerstens, T W J Huizinga, C F Allaart, W F Lems

**Affiliations:** 1 Department of Rheumatology, Leiden University Medical Center, PO BOX 9600, 2300 RC, Leiden, The Netherlands; 2 Franciscus Hospital, Roosendaal, the Netherlands; 3 MCH Haaglanden, Leidschendam, the Netherlands; 4 Jan van Breemen Research Institute | Reade, Amsterdam, the Netherlands; 5 VUMC, Amsterdam, the Netherlands

**Keywords:** Rheumatoid arthritis, Vertebral fractures, Bone mineral density, Functional ability, Disease activity, Treatment strategy

## Abstract

**Objective:**

To determine the prevalence of vertebral fractures (VFs) after 5 years of disease activity score (DAS)-steered treatment in patients with early rheumatoid arthritis (RA) and to investigate the association of VFs with disease activity, functional ability and bone mineral density (BMD) over time.

**Methods:**

Five-year radiographs of the spine of 275 patients in the BeSt study, a randomized trial comparing four treatment strategies, were used. Treatment was DAS-steered (DAS ≤ 2.4). A height reduction >20% in one vertebra was defined a vertebral fracture. With linear mixed models, DAS and Health Assessment Questionnaire (HAQ) scores over 5 years were compared for patients with and without VFs. With generalized estimating equations the association between BMD and VFs was determined.

**Results:**

VFs were observed in 41/275 patients (15%). No difference in prevalence was found when stratified for gender, prednisone use and menopausal status. Disease activity over time was higher in patients with VFs, mean difference 0.20 (95% CI: 0.05-0.36), and also HAQ scores were higher, independent of disease activity, with a mean difference of 0.12 (95% CI: 0.02-0.2). Age was associated with VFs (OR 1.06, 95% CI: 1.02-1.09), mean BMD in spine and hip over time were not (OR 95% CI, 0.99: 0.78-1.25 and 0.94: 0.65-1.36, respectively).

**Conclusion:**

After 5 years of DAS-steered treatment, 15% of these RA patients had VFs. Higher age was associated with the presence of VFs, mean BMD in hip and spine were not. Patients with VFs have greater functional disability over time and a higher disease activity, suggesting that VFs may be prevented by optimal disease activity suppression.

## Background

Vertebral fractures are more common in patients with RA compared to the general population [1-5]. Patients with fractures have a decreased functional ability and a higher mortality compared to patients without fractures [3,6-9]. In patients with RA, in addition to established risk factors such as lower body mass index (BMI), increased age, lower bone mineral density (BMD) and the use of corticosteroids, longer disease duration and more severe disease are associated with osteoporosis and vertebral fractures [1-5,7,10]. It is suggested that an appropriate control of disease may be protective of the quality and density of bone and thus prevent the development of vertebral fractures [11]. Such control of disease is generally more effectively achieved with disease activity score (DAS) steered treatment strategies [12,13] and more early with initial combination therapy with a synthetic disease-modifying antirheumatic drugs (DMARDs) and prednisone or anti tumor necrosis factor (anti-TNF) [14-17].

To investigate if such a treatment strategy results in less vertebral fractures, we conducted a cross-sectional analysis to determine the prevalence of vertebral fractures after 5 years of DAS-steered treatment in patients with early active RA, treated according to four different treatment strategies. In addition, we investigated the association of vertebral fractures with disease activity, functional ability and mean bone mineral density over time.

## Methods

### Patients

All measurements were performed in the setting of the BeSt study, a randomized clinical trial (Netherlands trial register, NTR265) comparing four different treatment strategies in DMARD-naïve patients who fulfilled the revised 1987 American College of Rheumatology (ACR) inclusion criteria for RA. The ethics committees of all participating centers approved the study protocol and patients gave their written informed consent.

Patients were treated according to a dynamic protocol starting with (1) sequential monotherapy, (2) step-up therapy, (3) initial combination therapy with tapered high-dose prednisone or (4) initial combination therapy including infliximab. Treatment adjustments were made using the disease activity score (DAS) every three months and treatment aimed at a DAS ≤2.4. All data was collected prospectively and more details on the BeSt study design were previously published [16,18].

### Assessment of vertebral fractures

Radiographs of the lateral thoracic and lumbar spine, taken after 5 years of follow-up, were available in 275 of the 508 patients. 434/508 patients were still under follow-up after 5 years of treatment and in 159/434 patients radiographs were not made, mostly due to logistic reasons in the various hospitals. This includes study personnel failing to send patients for radiographs and radiology personnel failing to make the radiographs as requested by protocol, but also some patients refused. Vertebral fractures were assessed with direct measurement and in consensus by two experienced readers (LD, WFL) using the Genant method. Deformities were classified as a wedge deformity, biconcave deformity or crush deformity. To quantify the severity of the fractures, the reduction in anterior, middle and/or posterior vertebral heights were measured and indicated as grade 1-3 deformities. Grade 1 represents a reduction in vertebral heights of 20-25%, grade 2 a reduction of 25-40% and grade 3 a reduction of more than 40%. Patients with a reduction of at least 20% (grade 1) in at least one vertebra were defined as fractured.

### BMD measurements

BMD measurements of the lumbar spine in antero-posterior view and/or the left total hip at baseline and yearly up to year 5 were available in 194 patients. Dual energy X-ray absorptiometry (DEXA) was available in eight participating centers, four of which used a Hologic QDR 4500 (Hologic, Waltham, MA, USA) and four used a Lunar DPX (Lunar, Madison, WI, USA). The mean BMD is calculated for each individual patient from measurements performed using the same device.

### Statistical analysis

Demographic and clinical baseline characteristics for patients with and without vertebral fractures were compared. Differences were tested using the chi-square test for categorical data and either the students T-test or Mann Whitney U test for continuous data, depending on the distribution of the tested variable.

Prevalence of vertebral fractures after 5 years of treatment was determined in the whole patient group as well as stratified for gender, menopausal status, use of corticosteroids and bisphosphonates and treatment strategy.

Using linear mixed models (LMM) with a Toeplitz covariance structure, DAS and HAQ scores (continuous outcome variables) over 5 years were compared for patients with and without vertebral fractures. In the model with DAS as outcome, estimates were adjusted for gender and baseline age, DAS and BMI. In the model with HAQ score as outcome, estimates were adjusted for baseline age, HAQ, BMI, gender and the DAS score over 5 years. Using generalized estimating equations (GEE) with an exchangeable covariance structure, the association between the presence of vertebral fractures (dichotomous outcome) and BMD was assessed. The association between the prevalence of vertebral fractures and BMD of the spine and the total hip were first analyzed univariately (crude OR) and next they were analyzed adjusted for year, age, BMI, postmenopausal status, use of bisphosphonates, vitamin D and/or calcium, HRT and the use of prednisone (adjusted OR).

With univariate regression analyses we tested baseline variables (age, gender, symptom duration, DAS, HAQ, BMI, erythrocyte sedimentation rate (ESR), C-reactive protein (CRP), RF, ACPA, BMD of the spine and BMD of the hip, smoking status and treatment strategy) for association with vertebral fractures after 5 years. Variables showing an association (*p* < 0.10) with vertebral fractures were entered as possible predictors in a multivariate logistic regression and significant independent predictors were identified with a backward selection procedure, using a p-value of 0.10 as the removal criterion. All tests were two-tailed and *p* < 0.05 was considered to be statistically significant. To analyze the data, SPSS version 17.0 software (SPSS, Chicago, IL, USA) was used.

## Results

Patients included in this study were on average 54 years old at baseline and most were female (67%), of whom 18% were postmenopausal. Rheumatoid arthritis at baseline was active with a mean DAS of 4.4 and a mean HAQ score of 1.3. Sixty-six and 63% of the patients were RF and ACPA positive, respectively (table 1).

**Table 1:** Baseline characteristics of the 275 out of 508 patients in the BeSt study with and without vertebral fractures after 5 years of treatment

**Baseline characteristics**	**Without vertebral fractures (n = 234)**	**With vertebral fractures (n = 41)**	**p-value**
Age, mean ± SD years	53 (13)	60 (11)	0.001
Female gender, no. (%)	159 (68)	26 (63)	0.568
Symptom duration, median (IQR) weeks	24 (14-50)	23 (15-49)	0.673
DAS, mean ± SD	4.4 (0.9)	4.2 (0.7)	0.069
HAQ, mean ± SD	1.3 (0.6)	1.4 (0.7)	0.594
BMI, mean ± SD	26 (4)	25 (4)	0.545
ESR, mean ± SD	36 (19-56)	44 (26-57)	0.098
CRP, mean ± SD	20 (9-52)	30 (12-51)	0.298
RF positive, no. (%)	152 (65)	29 (71)	0.472
ACPA positive, no. (%)	148 (64)	24 (59)	0.543
Smoking yes, no (%)	80 (34)	21 (51)	0.037
Treatment strategy, no. (%)			0.833
Sequential monotherapy	67 (28.6)	9 (22)	
Step-up therapy	48 (20.5)	10 (24.4)	
Initial combo with prednisone	55 (23.5)	10 (24.4)	
Initial combo with infliximab	64 (27.4)	12 (29.3)	

IQR, inter quartile range; DAS, disease activity score; HAQ, Health assessment Questionnaire; BMI, body mass index ; CRP, C-reactive protein; ESR, erythrocyte sedimentation rate; RF, rheumatoid factor; ACPA, anti-cyclic citrullinated peptide antibodies.

There were no significant differences in baseline characteristics (disease activity, age, BMI, gender, smoking and RF and ACPA status, symptom duration, ESR and CRP) between the 275 patients with radiographs of the spine and the patients without (data not shown), with the exception of baseline HAQ, which was lower in patients with radiographs of the spine (1.3 versus 1.5). Patients with BMD measurements had similar baseline characteristics as patients without, with the exception of BMI (25.5 versus 26.3 kg/m^2^) and HAQ score (1.3 versus 1.5).

### Prevalence of vertebral fractures

**Figure 1:**
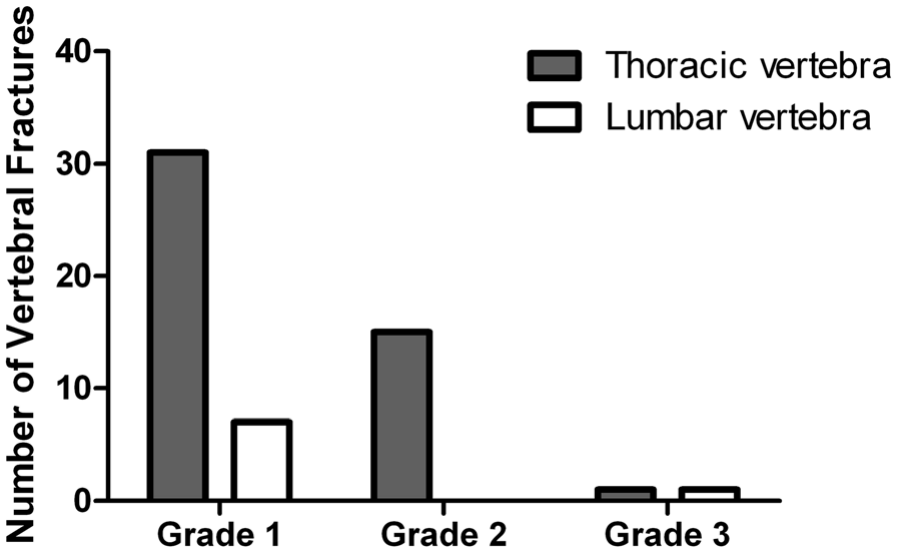
Number of vertebral fractures of the thoracic and lumbar vertebrae according the Genant method; Grade 1 represents a reduction in vertebral heights of 20-25%, grade 2 a reduction of 25-40% and grade 3 a reduction of more than 40%.

Vertebral fractures were observed in 41/275 patients (15%). Of the patients with fractures, 73% had one vertebral fracture, 22% had two vertebral fractures, 2.5% had three and 2.5% four vertebral fractures. In total, 55 of the 3691 vertebrae (1.5%) were fractured and most of these fractures were thoracic (85%). Most fractures were a grade 1 reduction (69%), 27% had a grade 2 reduction and 4% a grade 3 reduction (Figure 1).

Next, the prevalence of vertebral fractures was determined after stratifying for known risk factors. Patients with vertebral fractures after 5 years were older than patients without vertebral fractures (60 versus 53 years, *p* < 0.05) and were more often smokers (51% versus 34%, *p* < 0.05). The prevalence of vertebral fractures was similar in men and women (17% versus 14%, *p* = 0.568) and similar in pre- and postmenopausal women (9% versus 16%, *p* = 0.278). No difference in prevalence of vertebral fractures was found for the four different treatment groups (12, 17, 15 and 16% for group 1-4, respectively, *p* = 0.833). We found a prevalence of vertebral fractures of 19% in patients who ever used prednisone during 5 years of treatment and of 13% in those who had never used prednisone (*p* = 0.177). Similar results were found when patients were stratified for prednisone use more than one year versus prednisone use less than one year and when stratified for non-vertebral fractures (data not shown). Finally, use of bisphosphonates in 5 years time was similar in patients with or without vertebral fractures (25% versus 29%, *p* = 0.523) and similar results were found for use of calcium, vitamin D and hormone replacement therapy (data not shown).

### Vertebral fractures and BMD

**Figure 2:**
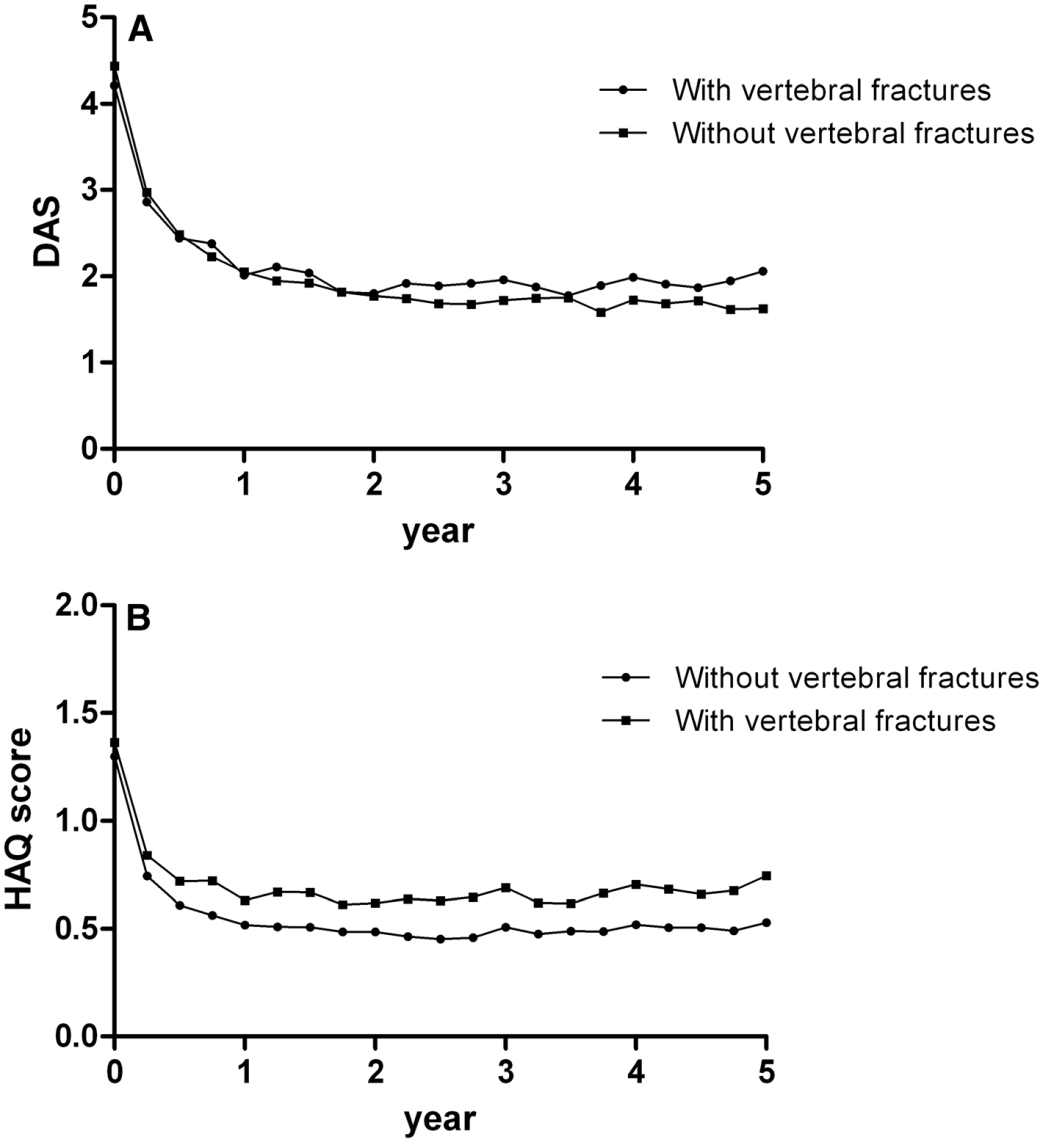
Bone mineral density (BMD) of the spine (figure A) and the total hip left (figure B) over 5 years time for patients with and without vertebral fractures.

Results of a Linear Mixed Models analysis show that the mean predicted BMD values of the spine were slightly lower over time in patients with vertebral fractures (1.03 versus 1.06 g/cm^2^, *p* = 0.283), while remaining stable over 5 years of treatment in both groups (Figure 2A). Mean predicted BMD values of the total hip were also slightly lower for patients with vertebral fractures (0.89 versus 0.93 g/cm^2^, *p* = 0.233), and decreased over time in patients with or without vertebral fractures (Figure 2B).

Mean BMD over time in the spine and mean BMD over time in the total hip were not significantly associated with the presence of vertebral fractures after five years of DAS-steered treatment (OR 0.86, 95% CI: 0.68-1.09 and OR 0.77, 95% CI: 0.54-1.11, respectively), also after adjustment for possible confounders (spine, OR 0.99, 95% CI: 0.78-1.25; hip, OR 0.94, 95% CI: 0.65-1.36). In the multivariate GEE models, only age was independently associated with the presence of vertebral fractures after 5 years of treatment with an OR (95% CI) of 1.04 (1.02-1.09) in the spine and an OR (95% CI) of 1.05 (1.01-1.10) in the hip.

### Predictors of vertebral fractures

In the univariate regression analysis and subsequently in the multivariate logistic regression analysis, baseline age and baseline smoking status were associated (*p* < 0.10) with having a vertebral fracture after 5 years of DAS-steered treatment (age OR 1.06, 95% CI: 1.02-1.09; smoking OR 2.5, 95% CI: 1.2-5.0).

### Vertebral fractures and the association with disease activity and functional ability

**Figure 3:**
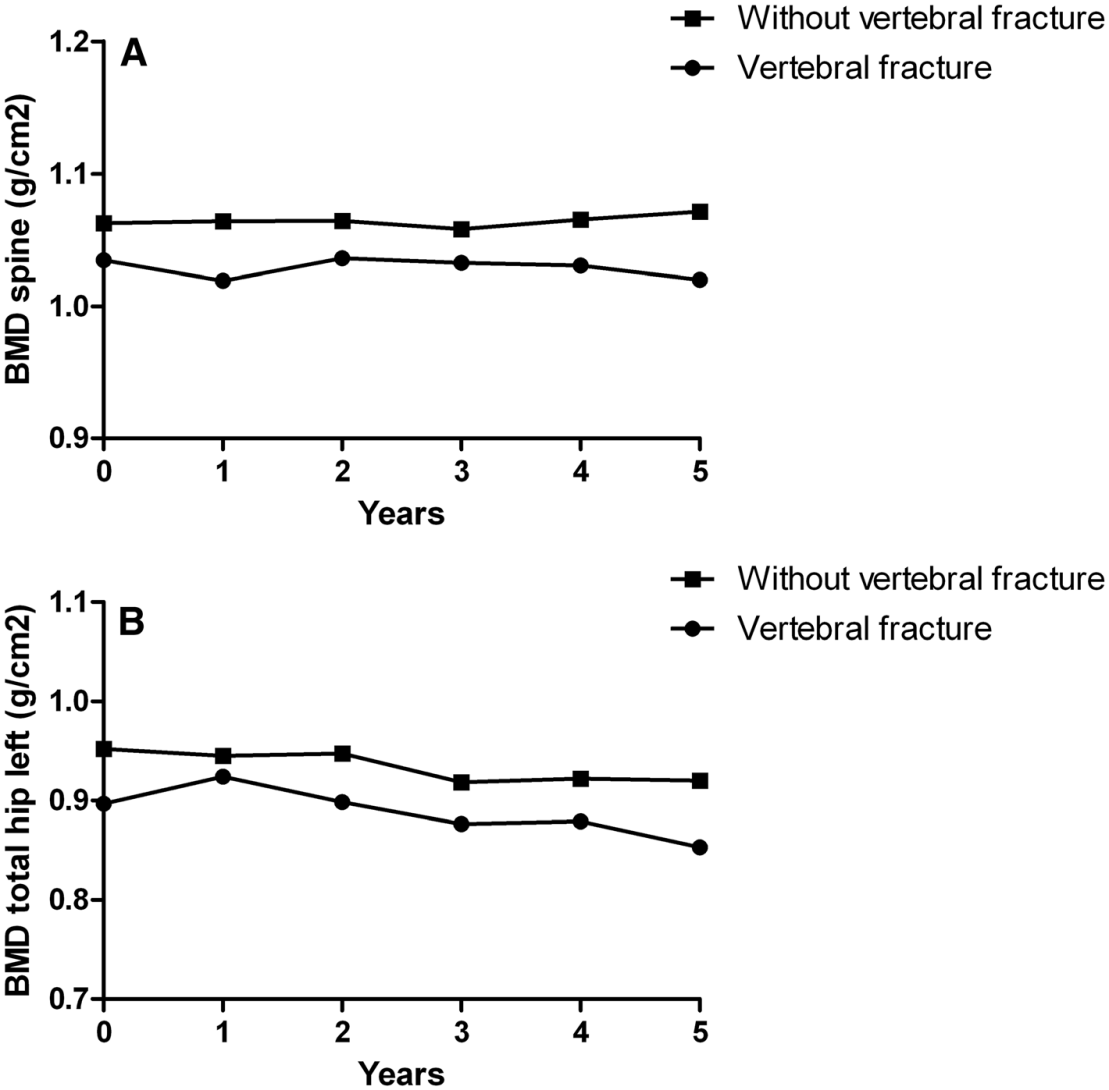
Disease activity scores (figure A) and HAQ scores (predicted scores based on a Linear Mixed Models analysis, corrected for concurrent DAS) (figure B) over 5 years time for patients with and without vertebral fractures.

In patients with vertebral fractures there was a trend for a lower baseline DAS (*p* = 0.069), but over 5 years time patients with vertebral fractures had a higher DAS over time compared to patients without vertebral fractures, with a mean difference of 0.20 (95% CI: 0.05-0.36) (Figure 3A). In addition, functional ability over time was worse in patients with vertebral fractures than in patients without vertebral fractures (unadjusted mean difference 0.15 (95% CI: 0.01-0.29) which was independent of disease activity over the same period (adjusted mean difference 0.12 (95% CI: 0.02-0.2) (Figure 3B).

## Discussion

In this longitudinal cohort of patients with early active RA we conducted a cross-sectional analysis and found a prevalence of vertebral fractures of 15% after 5 years of DAS-steered treatment. The majority of these patients (73%) had only one vertebral fracture and most deformities were grade 1 (69%).

The prevalence is lower than previously reported in non-DAS steered cohorts (19-36%) [1-3,7,10,19]. This suggests that adequate control of disease activity in earlier RA, with a treat-to-target approach, may protect against the occurrence of vertebral fractures. However, the prevalence of vertebral fractures in the general population is about 5% [2,10], which suggests that vertebral fractures may be a symptom of RA even if the disease activity appears relatively well controlled.

Most previous studies have focused on the prevalence of vertebral fractures in female RA patients, and postmenopausal women appeared to be especially at risk [6]. In a population-based cohort study of men and women older than 55 years, the incidence of vertebral fractures was higher in women than in men with a decrease in BMD as an independent predictor [20]. We found that the prevalence of vertebral fractures in male patients in our cohort was not statistically different from the prevalence in female patients (17% versus 14%, respectively). The prevalence of vertebral fractures in males was similar to that in postmenopausal women (16%) and higher than in premenopausal females (9%). This is probably related to the fact that males were of similar age as the postmenopausal females (57 versus 58 years, *p* = 0.281). We found higher age to be independently associated with a high prevalence of vertebral fractures. Also smoking was a risk factor for vertebral fractures.

In contrast to other studies, we found no association with the use of prednisone [3,6,10]. No difference was found in vertebral fractures between the four treatment groups, where in group 3 patients were initially treated with a high dose of prednisone (60 mg daily, tapered in 7 weeks to 7.5 mg daily). Ever use of prednisone was not associated with more vertebral fractures compared to never use, nor was use of prednisone longer than a year compared with use of prednisone less than a year. This suggests that the beneficial effects of prednisone on suppression of disease activity outweigh the potentially deleterious effects on bone quality and vertebral fractures [11,21].

In this cohort, use of bisphosphonates appeared not to be associated with a lower prevalence of vertebral fractures. This is at odds with previous reports that the use of bisphosphonates reduces the risk of vertebral and non-vertebral fractures [21-23]. In daily clinical practice, bisphosphonates are only prescribed if risk factors for bone mineral density loss are present, implying confounding by indication. In addition, bisphosphonates could have been started after a fracture has occurred, which also might explain why we found no protective effect. Most important independent risk factor of vertebral fractures after 5 years was a higher age.

It has been demonstrated that lower BMD is associated with the presence of vertebral fractures. [3,10] We previously reported that BMD loss in the BeSt study was not depending on prednisone use but on suppression of disease activity [22]. Since also rheumatic disease activity over time was slightly higher in patients with vertebral fractures compared to patients without vertebral fractures (mean difference 0.19), these findings support the hypothesis that there is a relation between disease activity and poor vertebral bone quality. We did not find a statistically significant association between BMD and vertebral fractures, although mean BMDs over time of the spine and hip of patients with vertebral fractures were somewhat lower than patients without vertebral fractures. Probably, this is related to the small changes in BMD over time, as a result of adequate disease control. In addition, after adjustment for age, itself significantly associated with a higher prevalence of vertebral fractures, the association between BMD and vertebral fractures becomes smaller. It thus appears that age related factors play an important role in the development of vertebral fractures.

More of clinical importance, functional disability over time was higher in patients with vertebral fractures compared to those without. This association was earlier described in a cross-sectional study, which sheds no light on the possible mechanism behind it [24]. We found that the higher functional disability in patients with vertebral fractures was independent of disease activity and age, suggesting that patients with vertebral fractures suffer functional impairment due to the vertebral fractures or the underlying bone condition, independent of rheumatic symptoms. It may also be possible that HAQ deterioration occurred before the vertebral fractures, but as HAQ deterioration is dependent on disease activity in the early phases of RA [25] and as we found that patients with vertebral fractures have higher HAQ over time irrespective of DAS over time, it seems unlikely that HAQ had preceded vertebral fractures, as earlier proposed [24]. Therefore, prevention of vertebral fractures is important in terms of reducing functional disability.

A limitation of this study is the absence of baseline radiographs of the spine. We are therefore not sure when the vertebral fracture occurred in relation to the onset of RA and the start of antirheumatic and anti-resorptive treatment. It is possible that fractures occurred before RA was diagnosed, in which case the prevalence of vertebral fractures in well suppressed RA is even less than we observed, or that bisphosphonates were started after a fracture has occurred. Although the prevalence of vertebral fractures was determined cross-sectionally, the differences in disease activity over time between patients with and without vertebral fractures do suggest that inflammation plays a role in the occurrence of vertebral fractures.

In conclusion, in a cohort of patients with recent-onset active RA, after 5 years of DAS-steered treatment the prevalence of vertebral fractures was with 15% higher than in the healthy population and similar for males and females. Independent predictors for vertebral fractures were age and smoking. Mean BMD over time of the spine and hip were not associated with the presence of vertebral fractures and vertebral fractures did not occur more in patients who used prednisone than in patients who did not. Patients with vertebral fractures suffer greater functional disability over time than patients without, independent of a slightly higher rheumatic disease activity. This indicates that vertebral fractures can be seen as bony damage of rheumatoid inflammation and may be prevented by optimal disease activity suppression.

## Competing interests

'The authors declare that they have no competing interests.

## Authors’ contribution

LD performed the statistical analysis and interpreted the data and drafted the manuscript. MB was involved in analyzing and interpreting the data and revised the manuscript. JG, and WB contributed in the acquisition of the data and were involved in revising the manuscript. PK, TH and WL participated in the study design, contributed in the acquisition of the data and were involved in revising the manuscript by critically looking at the content. CA participated in the study design, contributed in the acquisition of the data and was involved in analyzing and interpreting the data and helped to draft the manuscript. All authors read and approved the final version of the manuscript.

## **Netherlands trial register**: NTR265

## Funding

The BeSt study was supported by the Dutch College of Health Insurances. Schering-Plough and Janssen Biologics B.V. provided additional funding. The authors were responsible for the study design, the collection, analysis and interpretation of all data, the writing of this article, and the decision to publish.Plough and Janssen Biologics B.V. provided additional funding. The authors were responsible for the study design, the collection, analysis and interpretation of all data, the writing of this article, and the decision to publish.

## Pre-publication history

The pre-publication history for this paper can be accessed here:

http://www.biomedcentral.com/1471-2474/13/125/prepub
